# A Collection of Target Mimics for Comprehensive Analysis of MicroRNA Function in *Arabidopsis thaliana*

**DOI:** 10.1371/journal.pgen.1001031

**Published:** 2010-07-22

**Authors:** Marco Todesco, Ignacio Rubio-Somoza, Javier Paz-Ares, Detlef Weigel

**Affiliations:** 1 Department of Molecular Biology, Max Planck Institute for Developmental Biology, Tübingen, Germany; 2 Department of Plant Molecular Genetics, Centro Nacional de Biotecnología–Consejo Superior de Investigaciones Científicas, Madrid, Spain; The University of North Carolina at Chapel Hill, United States of America

## Abstract

Many targets of plant microRNAs (miRNAs) are thought to play important roles in plant physiology and development. However, because plant miRNAs are typically encoded by medium-size gene families, it has often been difficult to assess their precise function. We report the generation of a large-scale collection of knockdowns for *Arabidopsis thaliana* miRNA families; this has been achieved using artificial miRNA target mimics, a recently developed technique fashioned on an endogenous mechanism of miRNA regulation. Morphological defects in the aerial part were observed for ∼20% of analyzed families, all of which are deeply conserved in land plants. In addition, we find that non-cleavable mimic sites can confer translational regulation in cis. Phenotypes of plants expressing target mimics directed against miRNAs involved in development were in several cases consistent with previous reports on plants expressing miRNA–resistant forms of individual target genes, indicating that a limited number of targets mediates most effects of these miRNAs. That less conserved miRNAs rarely had obvious effects on plant morphology suggests that most of them do not affect fundamental aspects of development. In addition to insight into modes of miRNA action, this study provides an important resource for the study of miRNA function in plants.

## Author Summary

MiRNAs are small RNA molecules that play an important role in regulating gene function, both in animals and in plants. In plants, miRNA target mimicry is an endogenous mechanism used to negatively regulate the activity of a specific miRNA family, through the production of a false target transcript that cannot be cleaved. This mechanism can be engineered to target different miRNA families. Using this technique, we have generated artificial target mimics predicted to reduce the activity of most of the miRNA families in *Arabidopsis thaliana* and have observed their effects on plant development. We found that deeply conserved miRNAs tend to have a strong impact on plant growth, while more recently evolved ones had generally less obvious effects, suggesting either that they primarily affect processes other than development, or else that they have more subtle or conditional functions or are even dispensable. In several cases, the effects on plant development that we observed closely resembled those seen in plants expressing miRNA–resistant versions of the major predicted targets, indicating that a limited number of targets mediates most effects of these miRNAs. Analyses of mimic expressing plants also support that plant miRNAs affect both transcript stability and protein accumulation. The artificial target mimic collection will be a useful resource to further investigate the function of individual miRNA families.

## Introduction

MicroRNAs (miRNAs) are a class of small RNA (sRNA) molecules that has recently emerged as a key regulator of gene activity. In plants, miRNAs are released from larger precursors (pri-miRNAs) in the nucleus mainly, by DICER-LIKE1 (DCL1) [1]. The resulting sRNA duplex is methylated and translocated to the cytoplasm where it can be loaded into an RNA-induced silencing complex (RISC) that includes a member of the ARGONAUTE (AGO) family as catalytic component. The RISC can then recognize mRNAs containing sequences complementary to the loaded miRNA [2]. In plants, cleavage of the target mRNA is an important mechanism for plant miRNA action, but there are also direct effects on protein accumulation, as reported for many animal miRNAs [3]–[11].

The spatio-temporal expression pattern of miRNA genes is regulated to a large extent at the transcriptional level, and different members of a miRNA family can have distinct, specialized expression domains [12]–[17]. An additional layer of regulation in miRNA action has been reported by Franco-Zorrilla and colleagues [18]. *IPS1* (*INDUCED BY PHOSPHATE STARVATION 1*) encodes a non-coding RNA with a short motif that is highly complementary to the sequence of miR399, which like *IPS1* is involved in the response to phosphate starvation [19]–[23]. In contrast to regular miRNA target sites, the *IPS1* sequence contains a three-nucleotide insertion in the center, corresponding to the position where normally miRNA-guided cleavage takes place, and this bulge in the miRNA/target pair prevents endonucleolytic cleavage of *IPS1* transcripts. This results in sequestration of RISC^miR399^, leading to a reduction of miR399 activity. A similar phenomenon, negative regulation of small RNA activity by a partially complementary mRNA, has been recently described in bacteria as well [24], [25].

MiRNA target mimicry can be exploited to study the effects of reducing the function of entire miRNA families [18]. Simultaneous inactivation of all miRNA family members by constructing multiply mutant lines has so far been achieved for only two relatively small families [16], [26]. Plant target mimics are conceptually similar to miRNA sponges, used to reduce miRNA activity in animals. MiRNA sponges are transcripts containing multiple miRNA binding sites that compete with endogenous target mRNAs, thereby reducing the efficiency of the corresponding miRNA [27]. Although in animals perfect-match miRNA binding sites seems sufficient to sequester miRNAs [28], such optimal sites would be generally cleaved in plants, and they would not succeed in sequestering the miRNA-loaded RISC. Consistent with this, plants overexpressing non-modified versions of miR156 and miR319 target genes show much milder phenotypes than plants expressing the corresponding target mimics [18], [29], [30]. Modifications of the miRNA binding site that prevent cleavage but still allow miRNA binding are therefore required to reduce miRNA activity in plants.

Here, we present a collection of transgenic plants expressing artificial target mimics designed to knockdown the majority of *Arabidopsis thaliana* miRNA families. One fifth of these lines have obvious morphological defects, which is in the same range as the approximately 10% of miRNA knockouts that caused phenotypic abnormalities or lethality in *Caenorhabditis elegans* [31]. We found a clear correlation between the evolutionary conservation of plant miRNA families and their effect on aerial plant morphology.

## Results/Discussion

### Design of target mimics

We generated artificial target mimics for 73 different families or subfamilies of miRNAs and expressed them in *Arabidopsis thaliana* plants under the control of the constitutive 35S CaMV promoter. As described [18], we modified the 23 nucleotide, miR399-complementary motif in *IPS1*. The different constructs, and the corresponding transgenic lines, are named “*MIM*”, followed by the numeric identifier of the targeted miRNA family or subfamily. We targeted all miRNA families reported in miRBase (http://microrna.sanger.ac.uk/sequences/index.shtml) and ASRP (http://asrp.cgrb.oregonstate.edu) [32] at the beginning of 2007, plus some of the miRNAs described subsequently [33]. The majority of the analyzed families have only been described in *Arabidopsis thaliana* and *Arabidopsis lyrata* [34], [35]. The remaining families are shared with other angiosperms, and less than a quarter has been detected in non-flowering plants, including gymnosperms, ferns or mosses [32], [33], [36], [37]. A complete list of *MIM* constructs, and the primer pairs used to generate them, can be found in Table S1. For miRNA target predictions, see [8], [33], unless stated otherwise.

A single artificial target mimic could be designed for most miRNA families. The mature miRNAs produced by members of the miR169 and miR171 families differ slightly, and different target mimics were designed for these subfamilies. Two target mimics were also designed for the miR161 family, which produce two mature miRNAs that have only partially overlapping sequences, and that target similar subsets of the *PPR* gene family [38]. Conversely, some miRNA families have very similar sequences and overlapping in vivo targets (e.g., miR159/319, miR156/157 and miR170/171a), and artificial target mimics might not be able to unambiguously discriminate between different miRNAs.

**Figure 1:**
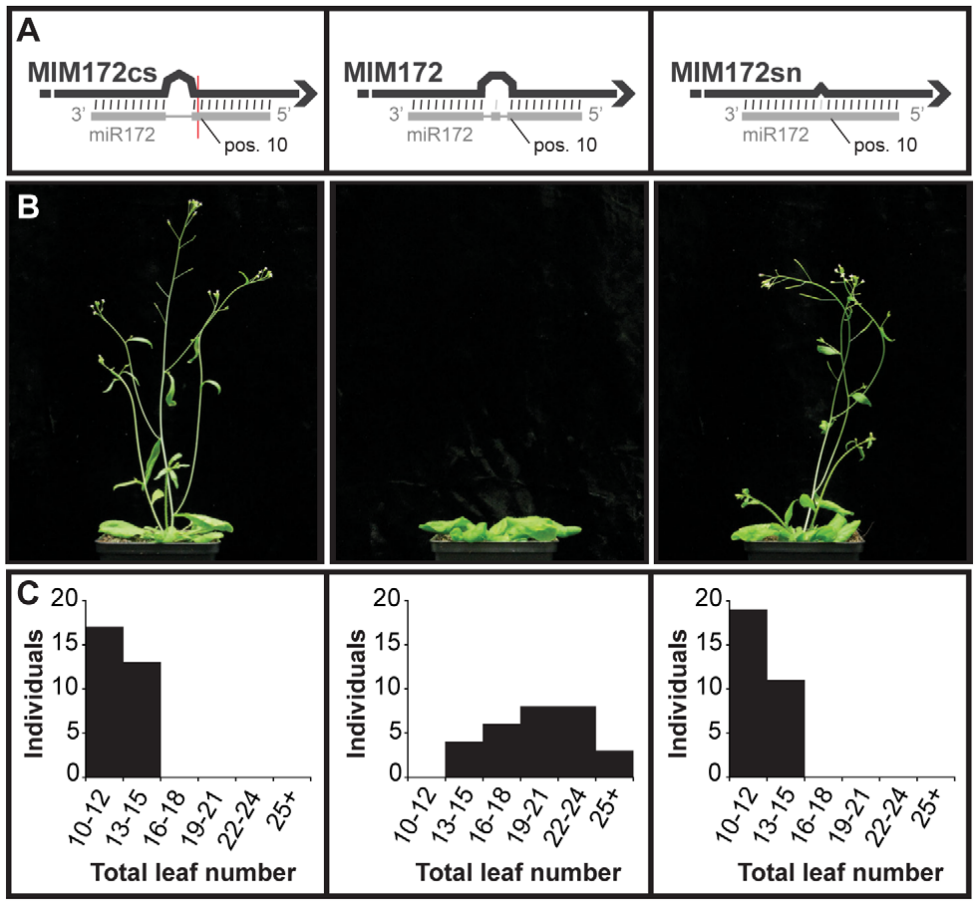
Requirement of a bulge at the cleavage site for target mimicry. (A) A target mimic with an unmodified central sequence (*MIM172cs*), which retained complementarity to the central portion of miR172 across the cleavage site (red line) opposite position 10 to 11 of the miRNA, did not change flowering time. Modification of the central sequence (TCTA to GAGT; *MIM172*) restored a three nucleotide bulge found in *IPS1* and generated a functional target mimic, causing a delay in flowering. However, a single nucleotide mismatch introduced into the center of an authentic miR172 target site (*MIM172sn*), but without a bulge, was not sufficient to reduce miR172 activity. (B) Four-week old plants grown at 23°C in long days. *MIM172cs* and *MIM172sn* are phenotypically indistinguishable from wild-type Col-0 plants (see also Figure S1B). (C) Distribution of flowering times of primary transformants grown in the same conditions; compare with Col-0 plants transformed with an empty binary vector in Figure S2.

In some cases, the sequence of the bulge in the miRNA/target mimic pair had to be modified. For example, maintaining the original central sequence of *IPS1* in *MIM172* could have reconstituted a cleavage site for miR172. Consistent with such modifications being important, plants expressing the appropriately modified version of *MIM172* showed an altered phenotype (see below), whereas plants expressing an initial version of *MIM172* in which a putative miR172 cleavage site was present (*MIM172cs*) did not. Moreover, plants expressing a *MIM172* version with only a single-nucleotide mismatch corresponding to position 11 of the mature miRNA (*MIM172sn*) did not show any abnormal phenotype either, suggesting that the three-nucleotide bulge is required for target mimic activity (Figure 1).

### Effects of target mimics on morphology and development

We generated at least 20 independent transformants for each of 75 separate constructs. Of these, 15, targeting 14 different families, caused reproducible phenotypes in the shoot system of the plants, which are described below. Phenotypic alterations were consistent across most, if not all, independent transformants examined for each construct. An example of the phenotypic variation among primary transformants is shown in the histograms in Figure 1. An overview of all lines with morphological defects is given in Table 1, together with the main target genes of the corresponding miRNA family and a list of other taxa in which they can be found. The phenotypes of *MIM156* and *MIM319* plants have been briefly described before [18], [39]. All miRNA families whose inactivation resulted in visible phenotypical alterations are conserved among the angiosperms, and most of them are also found in non-flowering plants.

**Table 1:** Artificial target mimics causing visible phenotypes.

Mimic	miRNAs*	Phenotype	miRNA targets	Conservation**
*MIM156*	miR156	Longer plastochron. Altered morphology of cotyledons and true leaves.	*SPL2*, *SPL3*, *SPL4*, *SPL5*, *SPL6*, *SPL9*, *SPL10*, *SPL11*, *SPL13*, *SPL15*	1, 2, 3, 4
*MIM157*	miR157	Similar to *MIM156*.	*SPL2*, *SPL4*, *SPL5*, *SPL6*, *SPL9*, *SPL10*, *SPL11*, *SPL13*, *SPL15*	
*MIM159*	miR159	Reduced size and stature. Thicker, upward curled leaves. Incomplete development of sepals, petals and anthers.	*MYB33*, *MYB65*, *MYB81*, *MYB97*, *MYB101*, *MYB104*, *MYB120*, *DUO1*	1, 2, 3
*MIM160*	miR160	Smaller plants, with serrated and curled upward leaves.	*ARF10*, *ARF16*, *ARF17*	1, 3, 4
*MIM164*	miR164	Partially serrated leaves. Ectopic tissue growth in the developing fruit.	*NAC1*, *CUC1*, *CUC2*, *ANAC079, ANAC092*, *ANAC100*, *AT3G12977*	1
*MIM165/166*	miR165/miR166	Rounder leaves. Younger leaves cup-shaped, with an irregular surface.	*PHV*, *PHB*, *REV*, *ATHB-8*, *ATHB-15*	1, 2, 3
*MIM167*	miR167	Delayed flowering. Twisted leaves, rolled downward. Defects in anther and seed development.	*ARF6*, *ARF8*	1, 3
*MIM169*	miR169a–c, h–n	Reduced rosette size.	*HAP2A*, *HAP2B*, *HAP2C*, *AT1G17590*, *AT1G54160*, *AT3G20910*, *AT5G06510*	1
*MIM169defg*	miR169dd–g	Similar to *MIM169.*	*HAP2A*, *HAP2B*, *HAP2C*, *AT1G17590*, *AT1G54160*, *AT3G20910*, *AT5G06510*	
*MIM170*	miR170	Round leaves of pale green color. Anthesis defects, causing reduced fertility.	*AT2G45160*, *AT3G60630*, *AT4G00150*	
*MIM171a*	miR171a	Similar to *MIM170*.	*AT2G45160*, *AT3G60630*, *AT4G00150*	1, 2, 3, 4
*MIM172*	miR172	Delay in flowering time. Narrow leaves, mildly rolled downward. Reduced apical dominance.	*AP2*, *TOE1*, *TOE2*, *TOE3*, *SMZ*, *SNZ*	1
*MIM319*	miR319	Similar to *MIM159*. In some lines, leaves curled downward.	*TCP2*, *TCP3*, *TCP4*, *TCP10 TCP24*, *MYB33*, *MYB65*, *MYB81*, *MYB97*, *MYB104*, *MYB120*	1, 2, 3, 4
*MIM393*	miR393	Narrow leaves, curled downward.	*AFB2*, *AFB3*, *TIR1*, *GRH1*, *AT3G23690*	1
*MIM394*	miR394	Narrow leaves, curled downward.	*AT1G27340*	1

*If no letter is given, the entire family was targeted.

**The conservation of miRNA families in the following groups is reported: (1) Other dicots and monocots, (2) gymnosperms, (3) ferns, (4) mosses.

**Figure 2:**
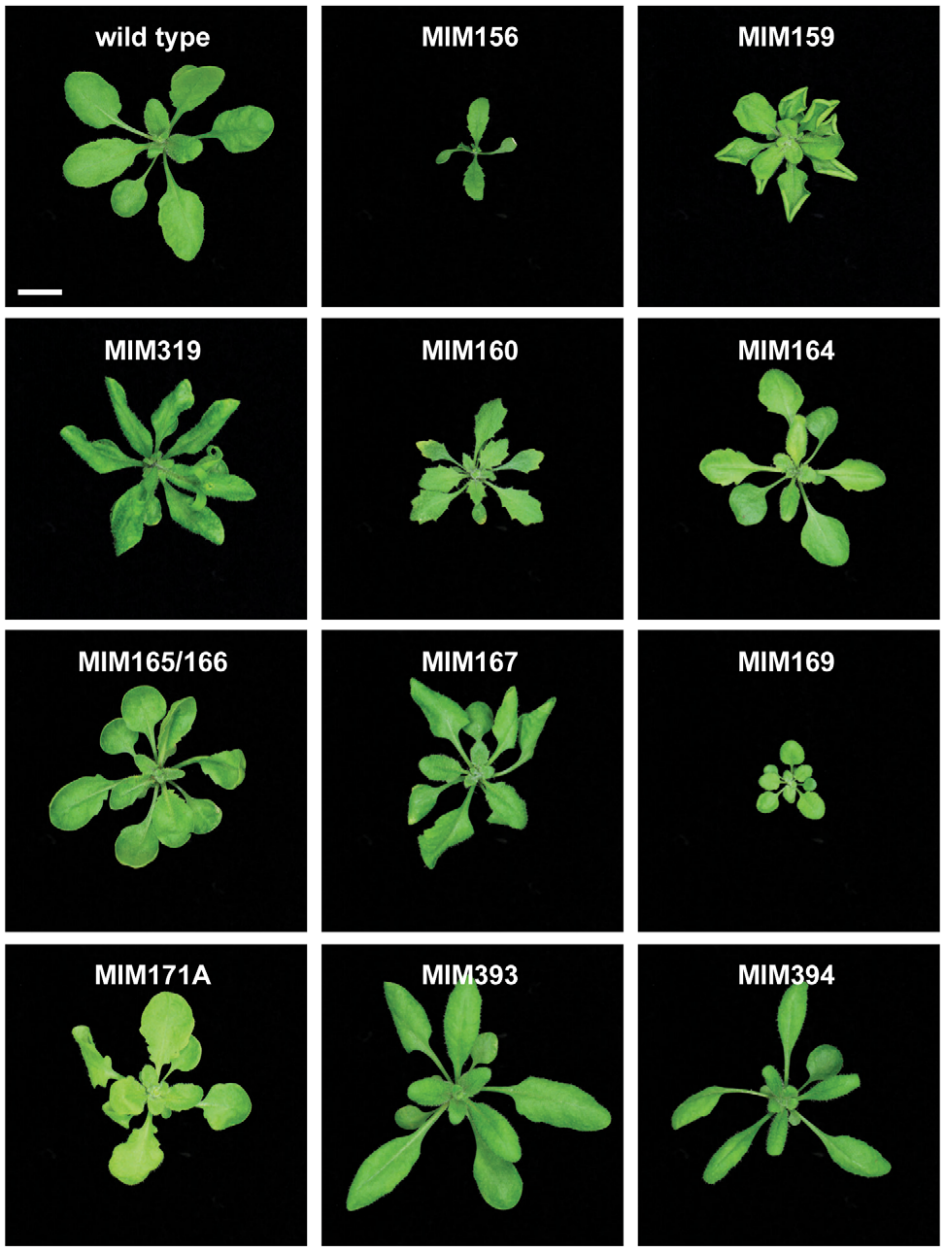
Leaf rosettes of target mimic expressing plants. Three-week-old plants. Bar corresponds to 1 cm for all panels.

*MIM156* and *MIM157* plants (Figure 2) had reduced leaf initiation rates, such that they flowered at about the same time as wild type, but with only two or three true leaves. This phenotype is similar to what is seen in plants carrying non-targetable versions of *SPL9* or *SPL10*, two of the miR156/157 targets, and opposite of plants overexpressing miR156b or *spl9 spl15* double mutants [10], [40]–[42]. In addition, these plants had bent, spoon-shaped cotyledons. The few rosette leaves were characterized by serrated margins, indicating adult leaf identity, consistent with a role of miR156 and its targets in controlling phase change [30].

**Figure 3:**
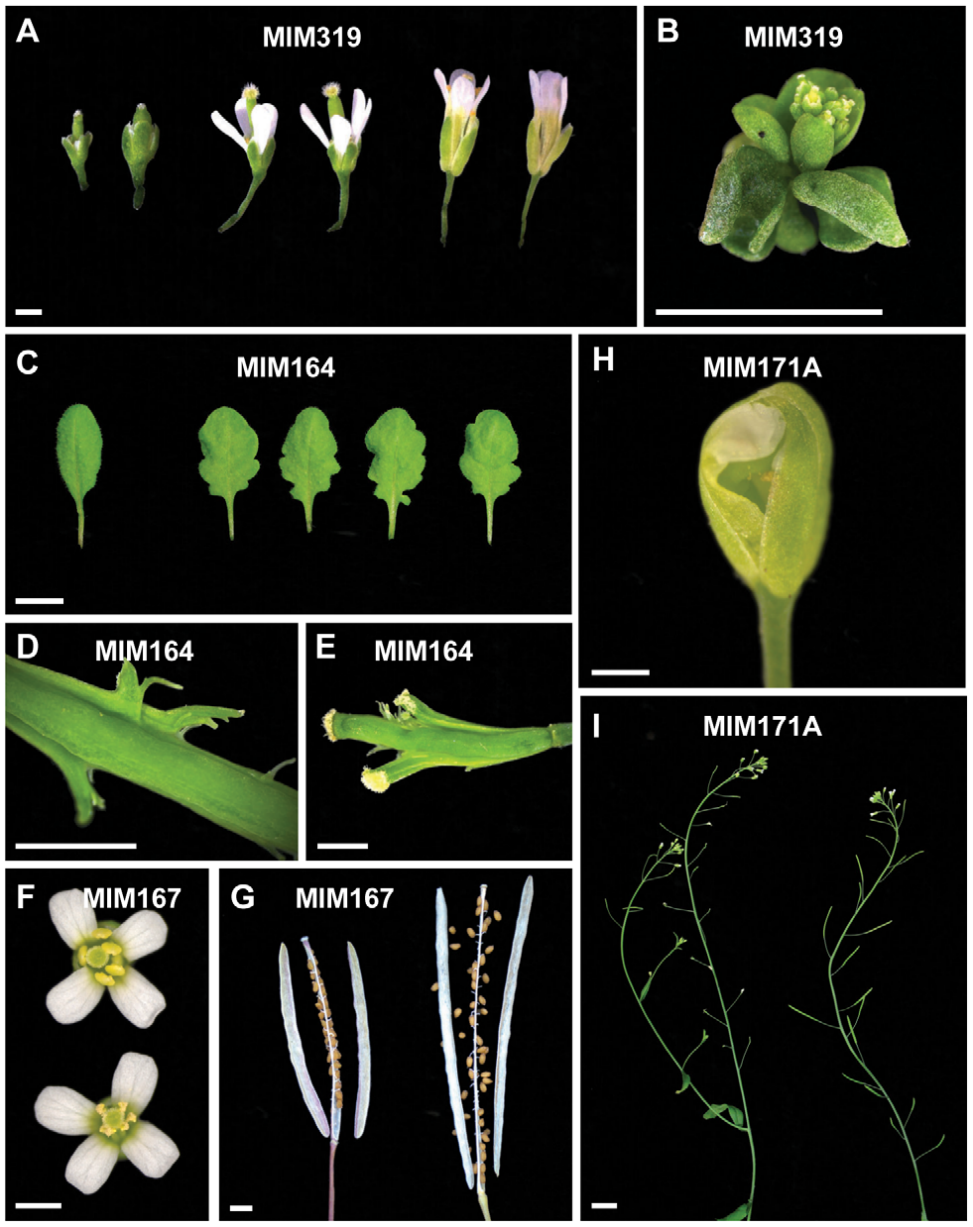
Details of defects observed in target mimic expressing plants. (A) Smaller flowers in severe *MIM319* lines. The most strongly affected flowers lacked petals and did not have fully developed anthers (left side); in milder lines, flowers had short sepals, narrow petals, but were fertile (middle). Two flowers from wild type Col-0 are shown on the right side of the panel. (B) Severe *MIM159* and *MIM319* lines were very small and compact, without any stem elongation. (C) Leaves of *MIM164* plants (compared to a leaf from wild type Col-0, on the far left). (D, E) Developing fruits of *MIM164* with ectopic growths emanating from valve margins (D), which can develop into pseudo-pistils in severe lines (E). (F) Anthers in *MIM167* lines did not mature completely (top), resulting in reduced pollen production (compared to a wild type Col-0 flower, bottom). (G) Seeds of *MIM167* plants often do not fill completely, and remained attached to the dried silique (compared to a silique of wild type Col-0, on the right). (H, I) *MIM171A* lines suffered from defects in the separation of sepals, which prevented emergence of the pistil (H), and caused the plants to be mostly sterile (I, on the left, compared to a wild-type Col-0 plant, on the right). Bars correspond to 1 cm in (A–C) and I, and to 0.1 cm in (D–H).

*MIM159* plants had extensive pleiotropic defects, and similar phenotypes were observed in most *MIM319* lines. These plants had reduced stature, with rounder, upward curled leaves (Figure 2), shorter stem internodes, and smaller flowers with short sepals, reduced petals and anthers that did not develop completely. More severe *MIM319* lines were progressively smaller, had warped leaves and lacked well-developed petals (Figure 3A). Stem elongation was often completely suppressed (Figure 3B). Most plants had reduced fertility, and this phenotype was particularly severe in *MIM319* plants, for which only a few viable seeds could be recovered after they were grown for prolonged periods at 16°C in long days. Both vegetative and floral phenotypes reminiscent of *MIM159* defects have been reported for plants that express non-targetable forms of miR159 target genes [29], and in plants doubly mutant for miR159a and miR159b [26]. In particular, upward curled leaves have been observed in plant expressing non-targetable forms of *MYB33*, which can be targeted both by miR159 and miR319 [43]. Milder *MIM319* lines showed different leaf defects, with leaves curled downward (Figure 2). This is consistent with what has been reported for plants that express non-targetable forms of *TCP2* and *TCP4*, which are both exclusive miR319 targets [29], suggesting that target mimics can at least partially discriminate between these two miRNA families.

Serrated and hyponastic leaves were seen in *MIM160* plants (Figure 2), in agreement with the phenotype of plants that express non-targetable versions of *ARF10* or *ARF17*, two of the three miR160 targets [44], [45]. In addition, *MIM160* plants were smaller than wild type. Compared to other constructs, fewer transformants were recovered, consistent with the known requirement of miR160 for seed viability or germination [44].

A different type of leaf serration was caused by *MIM164* (Figure 2), similar to what has been reported for plants expressing a non-targetable version of *CUC2*, one of the miR164 targets, and for plants lacking one of the miR164 isoforms, miR164a [13]. While expression of *MIM160* affected the entire leaf, with the serrations being regular and jagged, *MIM164* caused mainly serration of the basal part of the leaf, with more irregular and rounded sinuses and teeth (Figure 3C). Although carpel fusion defects have been described for plants lacking miR164c [12], the carpel defects in *MIM164* plants seemed to be different, with ectopic growths forming at the valve margins (Figure 3D), resembling those seen in the *cuc2-1D* mutant, in which a point mutation affects the miR164 complementary motif in *CUC2* [46]. In some cases, this tissue could develop into adventitious pistil-like structures (Figure 3E).

Rounder leaves with an irregular surface, which appeared to be hollowed out between the main veins, were caused by *MIM165/166*. Younger leaves tended also to be cup-shaped (Figure 2). Targets of miR165/166, including the transcription factor-encoding genes *PHAVOLUTA* and *PHABULOSA*, control leaf polarity, and dominant mutations that disrupt the miRNA target site in these genes cause severe alterations in leaf morphology [47]–[49].

A substantial delay in flowering was observed in *MIM167* plants, which flowered with 20.8±4.2 (mean ± standard deviation; *n* = 30) leaves in long days, compared to 13.0±0.9 rosette leaves in wild-type plants (Figure S1A and Figure S2). These plants had in addition twisted leaves (Figure 2), as well as defects in the maturation of anthers (Figure 3F) and in the development and shattering of seeds, which often remained attached to the dehiscent siliques (Figure 3G), resulting in reduced seed production and germination (not shown). This is consistent with what has been observed in plants that express a non-targetable form of the miR167 target *ARF6* or *ARF8*. Such plants have smaller leaves and are often sterile due to defects both in ovule and anther development [17]. Effects on flowering time have not been previously associated with miR167 [17], [50], and the late-flowering phenotype of *MIM167* plants reveals a new role for this miRNA family.

Two constructs were used to downregulate different subfamilies of miR169 family, whose main targets are *HAP* transcription factors. *MIM169* was designed for miR169a, b, c, h, i, j, k, l, m and n, and *MIM169defg* for miR169d, e, f and g. Both target mimics reduced the size of transgenic plants (Figure 2).

MiR170 and miR171 target a group of *SCARECROW*-like transcription factor genes [9], and both *MIM170* and *MIM171A* plants had round, pale leaves (Figure 2), as well as defective flowers, with sepals that did not separate properly, resulting in reduced fertility (Figure 3H and 3I). Expression of target mimics against the b and c members of the miR171 family did not confer any phenotype, suggesting less important roles for these two miRNAs.

*MIM172* plants were also late flowering, with 20.0±3.5 (*n* = 30) rosette leaves in long days (Figure S1B), consistent with the flowering time phenotype of plants that have increased expression of miR172 targets [4], [6], [51]. In addition, leaves of *MIM172* plants appeared to be somewhat narrower than those of wild type, and mildly curled downward, and severe *MIM172* lines presented reduced apical dominance (not shown). In contrast to plants that express a non-targetable version of *AP2* [52], flowers of *MIM172* plants were normal. These differential effects could be due to the particularly high levels of miR172 levels during early flower development [6].

MiR393 targets a small group of auxin receptor genes. *MIM393* plants had mild defects in leaf morphology, with narrow leaves that were curled downward (Figure 2). Leaf epinasty is often associated with high auxin levels [53], and is consistent with an increase of auxin signaling caused by downregulation of miR393 activity.

Finally, epinastic leaves were observed also in *MIM394* plants (Figure 2). MiR394 is predicted to target a gene encoding an F-box protein.

### Effects of target mimics on miRNA target genes

**Figure 4:**
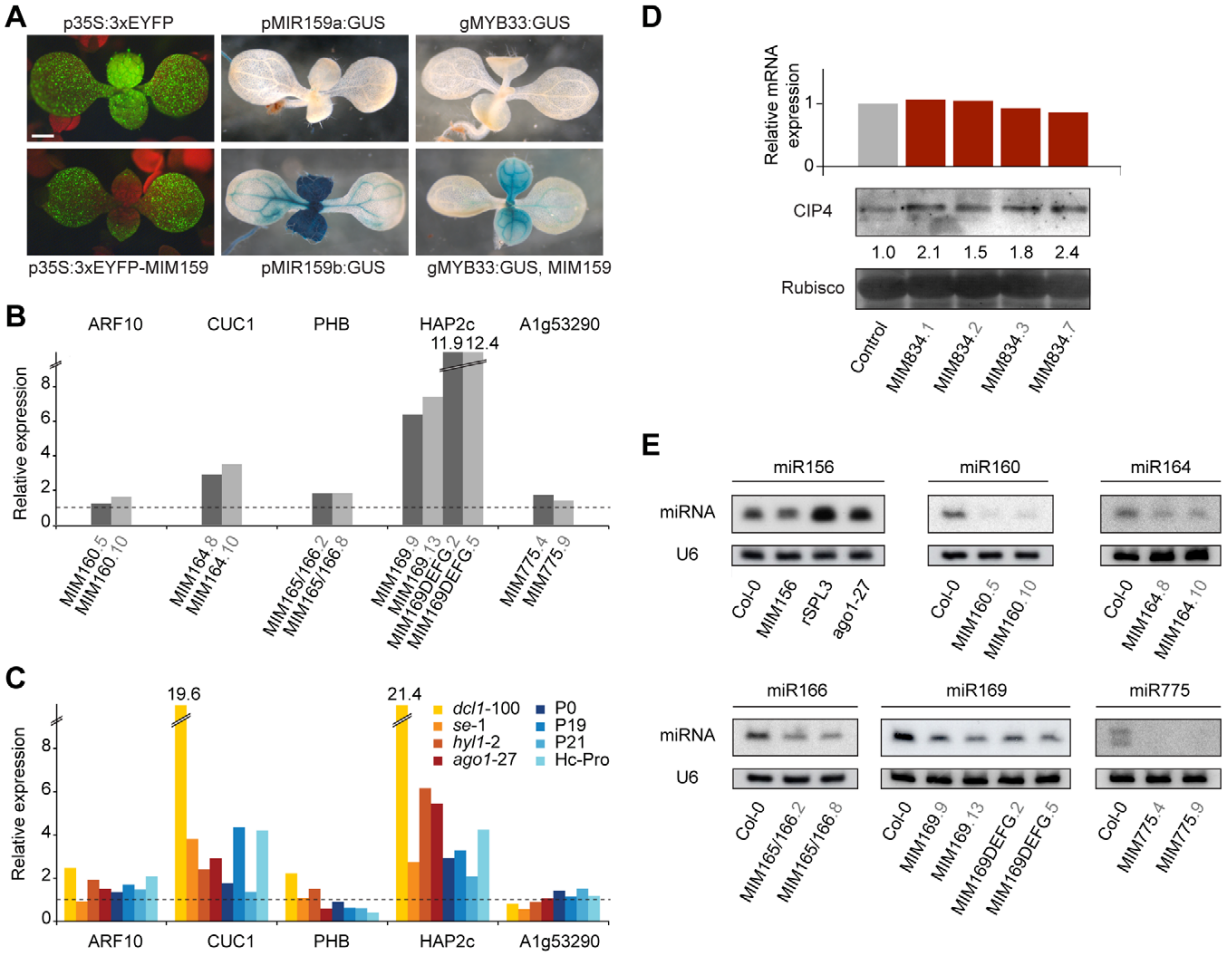
Effects of artificial mimics on levels of miRNAs and miRNA targets. (A) Nine-day-old plants. Introduction of a *MIM159* fragment into the 3′ UTR silences a constitutively expressed 3xEYFP in the *MIR159* expression domain (compare *p35S:3xEYFP* and *p35S:3xEYFP-MIM159*), which is revealed in the *pMIR159:GUS* lines. MiR159 activity is also indirectly revealed by comparing the effect of expressing *MIM159* in a genomic *MYB33:GUS* line. (B) Transcript levels of select miRNA targets in two independent lines for each *MIM* construct (represented by bars of different shades of gray). (C) Expression levels of miRNA targets in mutants impaired in miRNA biogenesis or targeting. Expression values are reported as the average of two biological and two technical replicates, and are normalized to the expression levels in wild type Col-0 plants (dotted line). (D) *CIP4* mRNA and protein levels in four independent *MIM834* lines. Band intensity relative to the wild-type control is reported. (E) Levels of mature miRNAs in several *MIM* lines. U6 accumulation is shown as control. Increased accumulation of miR156 (lower band in the blot) was observed upon expression of a resistant version of a miR156 target (consistent with what observed for miRNA156a precursor levels in [39]) or inhibition of miRNA activity in the *ago1*-27 mutants. The decrease in miR156 levels in *MIM156* plants is then not an indirect consequence of increased *SPL* transcript levels.

Artificial target mimics are thought to sequester their target miRNAs, presumably by stably binding to miRNA-loaded RISCs. To obtain additional evidence for such interactions, we embedded a functional *MIM159* site in the 3′-UTR of a triple- Enhanced Yellow Fluorescent Protein (EYFP) reporter; stable recruitment of RISC^miR399^ to the mimic site could be expected to interfere with EYFP translation. In 80% of *MIM159* expressing T1 plants, as in control plants, the EYFP transgene was completely silenced. In the remaining 20%, we detected EYFP signal that was strongly reduced in the region where *MIR159* genes are known to be expressed (Figure 4A) [26]. In addition, these plants presented the typical phenotypic defects of *MIM159* plants, confirming that the *EYFP:MIM159* construct functions properly as a target mimic.

RISC^miRNA^ sequestration in turn should relieve target genes from miRNA-dependent regulation, resulting in increased levels of the encoded protein. In agreement with such a scenario, activity levels of a genomic *MYB33:GUS* reporter were markedly increased in *MIM159* plants (Figure 4A). In analogy with *EYFP:MIM159*, reporter activity was increased in the tissues expressing *MIR159* genes [26], as expected.

Sequestration of RISC^miR399^ by the natural target mimic *IPS1* prevents miR399-guided cleavage of *PHO2* mRNA, thus increasing *PHO2* mRNA levels [18]. To assess the effects of artificial target mimics on the levels of mRNA of miRNA target genes, we tested them by reverse transcription followed by quantitative PCR (qRT-PCR) in a subset of *MIM* lines. We preferentially analyzed organs in which miRNA abundance was high according to the ASRP database [32], [54], or organs with major phenotypic alterations in *MIM* lines. Two independent lines were tested for each construct. Among the miRNA targets, we chose ones known to induce phenotypic defects when expressed as non-targetable forms [44], [45], [47] and ones that show altered expression in miRNA biogenesis mutants [32], [54], [55]. PCR products spanned the miRNA target sequence, allowing quantification of the attenuation in slicing activity by the corresponding miRNA. Surprisingly, in most cases there were no major changes in target transcript levels (Figure 4B and Figure S3).

For comparison, we examined the expression of the same miRNA target genes in seedlings of several mutants impaired in small RNA biogenesis and function, including *dcl1-*100, *se-*1, *hyl1-2* and *ago1-*27, and in plants overexpressing viral silencing suppressors that are known to counteract the action of the small RNA machinery, including P1/HC-Pro, P0, P19 and p21 [56]–[60]. In most cases, the changes seen in *MIM* lines correlated with those seen in miRNA biogenesis mutants. Stronger effects were observed only in *dcl1*-100 plants (Figure 4C). These results are consistent with what has been observed in microarray studies of miRNA biogenesis mutants, including other *dcl1* alleles, *se* and *hyl1* [55], .

As in animals, inhibition of translation is an important component of miRNA function in plants [4], [6], [11]. To test whether artificial mimics impact miRNA effects independent of changes in target transcript accumulation, we monitored the protein levels produced by *CIP4*, a gene that is regulated by miR834 through translational inhibition [5], [62]. In *MIM834* lines, CIP4 levels were appreciably increased, while *CIP4* mRNA levels were unchanged (Figure 4D). Direct effects on protein translation could explain the absence of a clear correlation between target mRNA levels and plant phenotype in plants expressing artificial target mimics.

Finally, we investigated the levels of mature miRNAs in plants expressing artificial target mimics. In all *MIM* lines we examined, levels of the targeted miRNA were decreased, suggesting that unproductive interaction of RISC^miRNA^ with a decoy affects miRNA stability (Figure 4E). Although such an effect has not been observed in case of the endogenous IPS1-miR399 interaction [18], a similar reduction in small RNA levels triggered by a target mimic has been reported in bacteria [24], [25].

### Conclusions

We have generated a collection of transgenic plants expressing artificial target mimics designed to reduce activity for most of the known miRNA families in *Arabidopsis thaliana*. Inhibiting the function of 14 out of 71 miRNA families with target mimics led to morphological abnormalities. All of these families belong to the more abundant and widely conserved miRNA families, which were the first ones to be discovered (Table 1). This agrees with results from experiments in which miRNAs were overexpressed, miRNA target genes were mutated, or miRNA genes were inactivated by conventional knockouts [reviewed in 63]. Together, these findings are consistent with the scenario of frequent birth and death of miRNA genes, with only a few becoming fixed early on during evolution because they acquired a relevant function in plant development [33], [36]. More recently evolved, species-specific miRNAs could instead play a role in adaptation to certain abiotic or biotic challenges, or have no discernable function at all. Some miRNAs are known to regulate physiological traits, and they do not cause morphological abnormalities under standard benign conditions [20], [21], [64]. Such conditional effects would have escaped our screen, as would have defects in the root system of the plant. Moreover, compared to expression of non-targetable forms of miRNA target genes, or miRNA loss-of-function mutants, the defects of *MIM* plants were often weaker. Examples are the absence of an altered floral phenotype in *MIM172* plants, which is seen in plants that express a non-targetable version of *AP2* under the control of normal regulatory sequences [52], or the extra-petals phenotype seen in *mir164c* mutants, but not in *MIM164* plants [12]. Another caveat is that some miRNAs might be required for embryonic development, in which case only lines with relatively weak expression of the artificial target mimic might have survived. Such limitations could be overcome by tissue-specific or inducible expression of target mimics. On the other hand, while artificial mimics increase levels of individual miRNA target genes less strongly than what can be achieved by expression of miRNA-resistant forms, mimics have the advantage that they affect all targets simultaneously [18]. Apart from translational regulation [3]–[7], another possibility for the absence of a clear correlation between phenotypic severity and change in mRNA levels of miRNA targets could be that many miRNAs affect their targets only in a small set of cells. In these cases, assaying expression in whole organs would obscure the effects of miRNA downregulation on mRNA levels.

It has recently been suggested that plant miRNAs could also repress the translation of target mRNAs that have only limited sequence complementarity, as often happens in animals [5]. Support for the existence of miRNA binding sites with reduced complementarity in plants comes from an analysis of miR398, which regulates *COPPER SUPEROXIDE DISMUTASE* (*CSD*) genes. Certain mutations in the miR398 complementary motif site reduced the effects of miR398 on *CSD* mRNA, but not on protein levels [3]. We have shown that mimic-like sites, when introduced into the 3′-UTR of a protein-coding gene, not only are active in sequestering the targeted miRNA, but can also reduce protein levels produced by the mRNA linked in cis. This reduction likely occurs at the translational level, since mimic sites are not subject to miRNA-dependent slicing [18]. This observation opens an intriguing scenario in which mRNAs containing mimic-like sites, or possibly other sites with reduced complementarity to miRNAs, are regulated by miRNAs exclusively through translational inhibition. A further level of complexity is added by such sites reducing the effects of an miRNA on other mRNA with a sliceable miRNA targeting motif, similarly to what has been recently proposed in animal systems [65].

Nevertheless, as pointed out before [43], miRNA overexpression and knockout of major target genes normally produce very similar phenotypes, and these are generally the opposite of what is seen in plants with reduced activity of the miRNA. These observations are supported by our finding of extensive similarities between phenotypes caused by target mimics and by expressing resistant forms of individual targets. We conclude that, at least for the instances in which developmental defects could be observed, target genes with extensive complementarity likely account for the majority of miRNA effects, but that in certain cases targets regulated solely through translational inhibition via diverged target sites might be important as well.

## Materials and Methods

### Plant material

Plants were grown on soil in long days (16 h light/8 hours dark) under a mixture of cool and warm white fluorescent light at 23°C and 65% humidity. The *se-*1, *ago1-*27, and *hyl1-*2 and *dcl1*-100 mutants have been described [66]–[69]. *MIM834* plants were grown on MS media plates supplemented with 1% sucrose for 14 days in long days at 23°C. Plants overexpressing viral proteins Hc-Pro, P0, P19 and P21 were a kind gift from the Carrington lab.

### Transgenic lines

Artificial target mimics were generated by modifying the sequence of the *IPS1* gene [18]. All target mimics constructs were placed behind the constitutive CaMV 35S promoter in the pGREEN vector conferring resistance to BASTA [70]. For the *MYB33:GUS* reporter, a *MYB33* genomic fragment was PCR amplified, cloned into the TOPO-PCR8 Gateway vector (Invitrogen), and recombined through LR clonase reaction into pGWB433 [71] to generate a GUS translational fusion. The *MIM159* construct was introduced into three independent *MYB33-GUS* T2 lines. A *MIM159* site was placed in the 3′-UTR of a triple-EYFP sequence linked to a fragment encoding a nuclear localization signal (NLS) and driven by a CaMV 35S promoter. Constructs were introduced into *A. thaliana* (accession Col-0) plants by *Agrobacterium tumefaciens*-mediated transformation [72].

### Histochemical assays

Nine-day-old seedlings from three independent T2 lines for all the GUS reporter backgrounds were fixed in acetone 90%. GUS activity was assayed as described [73].

### RNA analysis

Total RNA was extracted from 11-day old seedlings and 30-day old inflorescences (47 days for the *MIM172* lines), using TRIzol Reagent (Invitrogen). For *dcl-*100, 13-day old seedlings were collected, to obtain a similar developmental stage compared to the other plants. For real time RT-PCR, two biological replicates with tissue pooled from 8 to 10 plants were assayed from two independent *MIM* lines per miRNA family or subfamily. Complementary DNA was produced with the RevertAid First Strand cDNA Synthesis Kit (Fermentas), using as starting material 4 µg of total RNA that had been treated with DNase I (Fermentas). PCR was carried out in presence of SYBR Green (Invitrogen) and monitored in real time with the Opticon Continuous Fluorescence Detection System (MJR). Oligonucleotide primers are given in Table S2. Small RNA blots were performed on the same RNA used as template for real time RT-PCR, with DNA oligonucleotides as probes.

### Protein analysis

Proteins were extracted from four *MIM834* lines using a Tris buffer (50 mM Tris pH 7,5; 150 mM NaCl; 1 mM EDTA; 10% [v/v] Glycerol; 1 mM DTT; 1 mM Pefablock and 1 complete protease inhibitor cocktail [Roche]). Protein concentration was measured using a commercial Bradford assay (BioRad). 50 µg of raw protein extract per sample were resolved on an 8% acrylamide gel. Blotting and antibody incubation were performed as described [5], except that the secondary antibody was incubated for 8 hours at 4°C. Two biological replicates from 4 independent lines were analyzed. Band intensity was measured using the ImageJ software (http://rsbweb.nih.gov/ij/).

## Supporting Information

Figure S1 Flowering behavior of *MIM167* and *MIM172* plants. Five-week-old, long-day grown plants expressing *MIM167* (A) and *MIM172* (B) next to wild-type Col-0 plants on the left.
(0.89 MB PDF)

Figure S2 Flowering time of *MIM167* plants and Col-0 controls. Distribution of flowering times of primary transformants of plants transformed with *MIM167* or empty pGREEN binary vector, grown at 23°C in long days.
(0.09 MB PDF)

Figure S3 Expression of miRNA targets in inflorescences of *MIM* lines. Transcript levels of select miRNA targets in two independent lines for each *MIM* construct (represented by bars of different shades of gray). Expression levels are reported as the average of two biological and two technical replicates, and are normalized to the expression levels in wild-type Col-0 plants (dotted line).
(0.10 MB PDF)

Table S1 Oligonucleotide primers used to modify the IPS1 sequence in *MIM* constructs.
(0.11 MB PDF)

Table S2 Oligonucleotide primers for qRT-PCR.
(0.07 MB PDF)
