# Supplementary material for: A Collection of Target Mimics for Comprehensive Analysis of MicroRNA Function in Arabidopsis thaliana
Source: PLoS Genet. 2010 Jul 22;6(7):e1001031. doi: 10.1371/journal.pgen.1001031 (PMC2908682; doi:10.1371/journal.pgen.1001031)
Supplement: Figure S1 — Flowering behavior of MIM167 and MIM172 plants. Five-week-old, long-day grown plants expressing MIM167 (A) and MIM172 (B) next to wild-type Col-0 plants on the left. (0.89 MB PDF) [file pgen.1001031.s001.pdf]

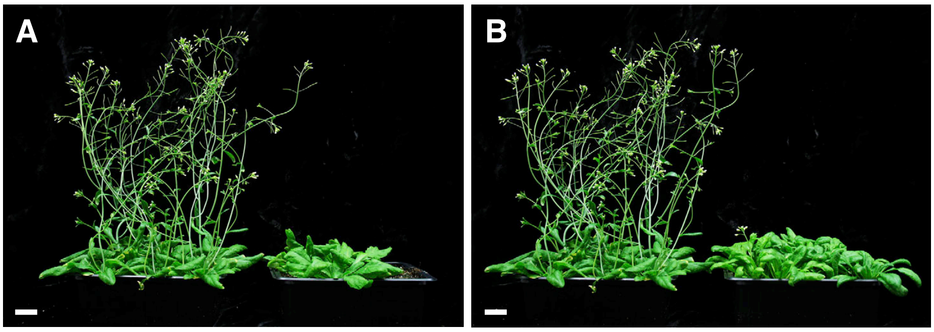

Supplementary Figure 1. Flowering behavior of *MIM167* and *MIM172* plants.

Five-week old, long-day grown plants expressing *MIM167* (A) and *MIM172* (B) next to wild type Col-0 plants on the left.
