# Supplementary material for: A Collection of Target Mimics for Comprehensive Analysis of MicroRNA Function in Arabidopsis thaliana
Source: PLoS Genet. 2010 Jul 22;6(7):e1001031. doi: 10.1371/journal.pgen.1001031 (PMC2908682; doi:10.1371/journal.pgen.1001031)
Supplement: Figure S2 — Flowering time of MIM167 plants and Col-0 controls. Distribution of flowering times of primary transformants of plants transformed with MIM167 or empty pGREEN binary vector, grown at 23°C in long days. (0.09 MB PDF) [file pgen.1001031.s002.pdf]

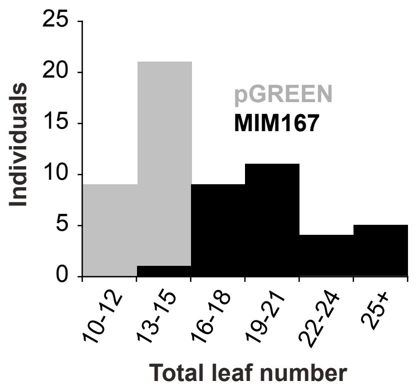

Supplementary Figure 2. Flowering time of *MIM167* plants and Col-0 controls.

Distribution of flowering times of primary transformants of plants transformed with *MIM167* or empty pGREEN binary vector, grown at 23°C in long days.
