# Supplementary material for: A Collection of Target Mimics for Comprehensive Analysis of MicroRNA Function in Arabidopsis thaliana
Source: PLoS Genet. 2010 Jul 22;6(7):e1001031. doi: 10.1371/journal.pgen.1001031 (PMC2908682; doi:10.1371/journal.pgen.1001031)
Supplement: Figure S3 — Expression of miRNA targets in inflorescences of MIM lines. Transcript levels of select miRNA targets in two independent lines for each MIM construct (represented by bars of different shades of gray). Expression levels are reported as the average of two biological and two technical replicates, and are normalized to the expression levels in wild-type Col-0 plants (dotted line). (0.10 MB PDF) [file pgen.1001031.s003.pdf]

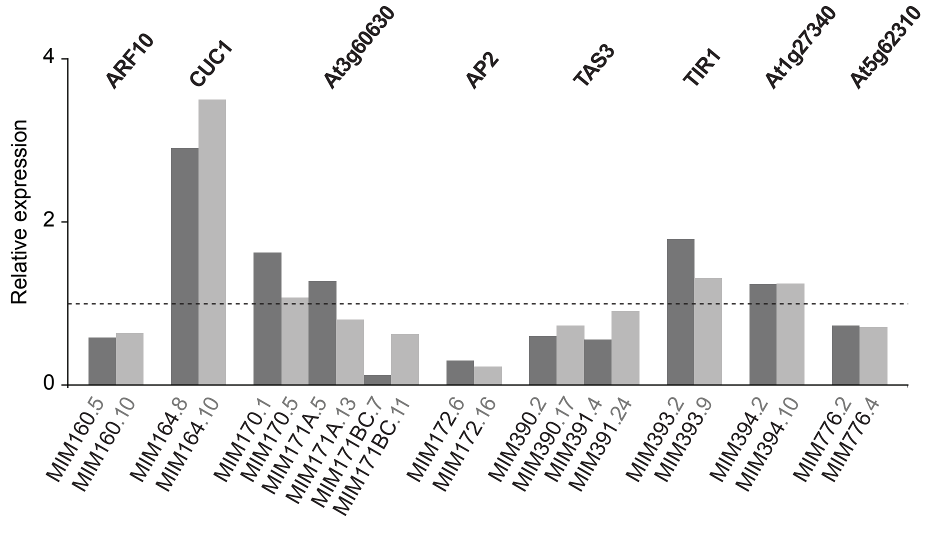

Supplementary Figure 3. Expression of miRNA targets in inflorescences of *MIM* lines.

Transcript levels of select miRNA targets in two independent lines for each *MIM* construct (represented by bars of different shades of gray). Expression levels are reported as the average of two biological and two technical replicates, and are normalized to the expression levels in wild type Col-0 plants (dotted line).
