# Supplementary material for: A Collection of Target Mimics for Comprehensive Analysis of MicroRNA Function in Arabidopsis thaliana
Source: PLoS Genet. 2010 Jul 22;6(7):e1001031. doi: 10.1371/journal.pgen.1001031 (PMC2908682; doi:10.1371/journal.pgen.1001031)
Supplement: Table S1 — Oligonucleotide primers used to modify the IPS1 sequence in MIM constructs. (0.11 MB PDF) [file pgen.1001031.s004.pdf]

Supplementary Table 1. Oligonucleotide primers used to modify the *IPS1* sequence in *MIM* constructs.

Construct	Primer	Sequence (5´- 3´)
MIM156	I	ctTGACAGAAGATAGAAGTGAGCATtttctagagggagataa
	II	aaATGCTCACTTCTATCTTCTGTCAagcttcggttcccctcg
MIM157	I	gaaccgaagctTTGACAGAAGTCGATAGAGAGCACtttctagagg
	II	cctctagaaaGTGCTCTCTATCGACTTCTGTCAAagcttcggttc
MIM158	I	ctTCGATAAACCAGATCTGCATCCAgtttctagagggagataa
	II	aaTGCTTTGTCTCAAACATTTGGGAagcttcggttcccctcg
MIM159	I	gaaccgaagctTTTGGATTGATTGAGGGAGCTCTTtttctagagg
	II	cctctagaaaAAGAGCTCCCTCAATCAATCCAAAagcttcggttc
MIM160	I	aaTGGCATACAGTCTAGAGCCAGGCAagcttcggttcccctcg
	II	ctTGCCTGGCTCTAGACTGTATGCCAtttctagagggagataa
MIM161a.1	I	gaaccgaagcTTGAAAGTGACGAACACATCGGGGttttctagagg
	II	cctctagaaaaCCCCGATGTGTTCGTCACTTTCAAgcttcggttc
MIM161a.2	I	gaaccgaagctTCAATGCATTTCTAAAAGTGACTAtttctagagg
	II	cctctagaaaTAGTCACTTTTAGAAATGCATTGAagcttcggttc
MIM162	I	ctTCGATAAACCAGATCTGCATCCAGtttctagagggagataa
	II	aaCTGGATGCAGATCTGGTTTATCGAagcttcggttcccctcg
MIM163	I	ctTTGAAGAGGATTGATTGGAACTTCGATtttctagagggagataa
	II	aaATCGAAGTTCCAATCAATCCTCTTCAAagcttcggttcccctcg
MIM164	I	ctTGGAGAAGCATAGGGGCACGTGCAtttctagagggagataa
	II	aaTGCACGTGCCCCTATGCTTCTCCAagcttcggttcccctcg
MIM165/166	I	ctTCGGACCAGGTAGATTCATTCCCCtttctagagggagataa
	II	aaGGGGAATGAATCTACCTGGTCCGAagcttcggttcccctcg
MIM167	I	gaaccgaagctTGAAGCTGCCTTGAGCATGATCTAtttctagagg
	II	ctctagaaaTAGATCATGCTCAAGGCAGCTTCAagcttcggttc
MIM168	I	ctTCGCTTGGTGTTTAAGGTCGGGAAtttctagagggagataa
	II	aaTTCCCGACCTTAAACACCAAGCGAagcttcggttcccctcg
MIM169	I	ctTAGCCAAGGAGCACGACTTGCCGAtttctagagggagataa
	II	aaTCGGCAAGTCGTGCTCCTTGGCTAagcttcggttcccctcg
MIM169defg	I	ctTGAGCCAAGGTAGGTGACTTGCCGtttctagagggagataa
	II	aaCGGCAAGTCACCTACCTTGGCTCAagcttcggttcccctcg
MIM170	I	ctTGATTGAGCCTTTATGTCAATATCtttctagagggagataa
	II	aaGATATTGACATAAAGGCTCAATCAagcttcggttcccctcg
MIM171a	I	ctTGATTGAGCCTTTACGCCAATATCtttctagagggagataa
	II	aaGATATTGGCGTAAAGGCTCAATCAagcttcggttcccctcg
MIM171bc	I	ctGTTAGCCGTGTTTACAATATCACGtttctagagggagataa
	II	aaCGTGATATTGTAAACACGGCTAACagcttcggttcccctcg

MIM172	I	ctAGAATCTTGAACTCGATGCTGCATtttctagagggagataa
	II	aaATGCAGCATCGAGTTCAAGATTCTagcttcggttcccctcg
MIM172cs	I	ctAGAATCTTGATAGAGATGCAGCTCtttctagagggagataa
	II	aaGAGCTGCATCTCTATCAAGATTCTagcttcggttcccctcg
MIM172ns	I	ccgaagctAGAATCTTGAGGATGCTGCATtttctagagg
	II	ctagaaaTGCAGCATCCTCAAGATTCTagcttcggttc
MIM173	I	gaaccgaagctTTCGCTTGCATTCGAGAGAAATCACtttctagagg
	II	cctctagaaaGTGATTTCTCTCGAATGCAAGCGAAagcttcggttc
MIM319	I	ctTTGGACTGAATAGAGGAGCTCCTtttctagagggagataa
	II	aaAGGAGCTCCTCTATTCAGTCCAAagcttcggttcccctcg
MIM390	I	ctAAGCTCAGGATAGAGGATAGCGCCtttctagagggagataa
	II	aaGGCGCTATCCTCTATCCTGAGCTTagcttcggttcccctcg
MIM391	I	ctTTCGCAGGAGTAGTGATAGCGCCAtttctagagggagataa
	II	aaTGGCGCTATCACTACTCCTGCGAAagcttcggttcccctcg
MIM393	I	ctTCCAAAGGGAGGGCCGCATTGATCCtttctagagggagataa
	II	aaGGATCAATGCGGCCCTCCCTTTGGAagcttcggttcccctcg
MIM394	I	ctTTGGCATTCTTAAGTCCACCTCCtttctagagggagataa
	II	aaGGAGGTGGACTTAAGAATGCCAAagcttcggttcccctcg
MIM395	I	ctCTGAAGTGTTAAGCGGGGAACTCtttctagagggagataa
	II	aaGAGTTCCCCGCTTAACACTTCAGagcttcggttcccctcg
MIM396	I	ctTTCCACAGCTAAGCTCTTGAACTGtttctagagggagataa
	II	aaCAGTTCAAGAGCTTAGCTGTGGAAagcttcggttcccctcg
MIM397	I	ctTCATTGAGTGTAAAATCGTTGATGtttctagagggagataa
	II	aaCATCAACGATTTTACACTCAATGAagcttcggttcccctcg
MIM398	I	ctTGTGTTCTCATTGAGTCACCCTGtttctagagggagataa
	II	aaCAGGGTGACTCAATGAGAACACAagcttcggttcccctcg
MIM400	I	gaaccgaagctTATGAGAGTAGAACTATAAGACActttctagagg
	II	cctctagaaaGTGTCTTATAGTTCTACTCTCATAagcttcggttc
MIM401	I	ctCGAAACTGGTTAGATCGACCGACAtttctagagggagataa
	II	aaTGTCGGTCGATCTAACCAGTTTCGagcttcggttcccctcg
MIM402	I	ctTTCGAGGCCTGGGATTAAACCTCTGtttctagagggagataa
	II	aaCAGAGGTTTAATCCCAGGCCTCGAAagcttcggttcccctcg
MIM403	I	ctTTAGATTCACTTGACACAAACTCGtttctagagggagataa
	II	aaCGAGTTTGTGTCAAGTGAATCTAAagcttcggttcccctcg
MIM404	I	ctATTAACGCTGTTCACGGTTGCGGCAGCtttctagagggagataa
	II	aaGCTGCCGCAACCGTGAACAGCGTTAATagcttcggttcccctcg
MIM405	I	ctATGAGTTGGGAACCCTAACCCATAACTtttctagagggagataa
	II	aaAGTTATGGGTTAGGGTTCCCAACTCATagcttcggttcccctcg
MIM406	I	ctTAGAATGCTAGAGCTGTAATCCAGtttctagagggagataa
	II	aaCTGGATTACAGCTCTAGCATTCTAagcttcggttcccctcg
MIM407	I	ctTTTAAATCATGGGGTACTTTTGGTtttctagagggagataa

	II	aaACCAAAAGTACCCCATGATTTAAAagcttcggttcccctcg
MIM408	I	ctATGCACTGCCAAGCCTTCCCTGGCtttctagagggagataa
	II	aaGCCAGGGAAGGCTTGGCAGTGCATagcttcggttcccctcg
MIM413	I	ctATAGTTTCTCAAGCTGTTCTGCACtttctagagggagataa
	II	aaGTGCAGAACAGCTTGAGAAACTATagcttcggttcccctcg
MIM414	I	ctTCATCTTCATTAGAATCATCGTCAtttctagagggagataa
	II	aaTGACGATGATTCTAATGAAGATGAagcttcggttcccctcg
MIM415	I	ctAACAGAGCAGTTCCAACAGAACATtttctagagggagataa
	II	aaATGTTCTGTTGGAACTGCTCTGTTagcttcggttcccctcg
MIM416	I	ctGGTTCGTACGAAGAACACTGTTCAtttctagagggagataa
	II	aaTGAACAGTGTTCTTCGTACGAACCagcttcggttcccctcg
MIM417	I	ctGAAGGTAGTGTATAATTTGTTCGAtttctagagggagataa
	II	aaTCGAACAAATTATACACTACCTTCagcttcggttcccctcg
MIM418	I	ctTAATGTGATGTTTATGAACTGACCtttctagagggagataa
	II	aaGGTCAGTTCATAAACATCACATTAagcttcggttcccctcg
MIM419	I	ctTTATGAAGGCAAGAGAGGATGTTGtttctagagggagataa
	II	aaCAACATCCTCTCTTGCCTTCATAAagcttcggttcccctcg
MIM420	I	ctTAAACTAATCTTGGCGGAAATGCAtttctagagggagataa
	II	aaTGCATTTCCGCCAAGATTAGTTTAagcttcggttcccctcg
MIM426	I	ctTTTTGGAAATAAGAGGTCCTTACGtttctagagggagataa
	II	aaCGTAAGGACCTCTTATTTCCAAAAagcttcggttcccctcg
MIM427	I	ctTTTTTCCTACAAGACCGCCCATACCtttctagagggagataa
	II	aaGGTATGGGCGGTCTTGTAGGAAAAAagcttcggttcccctcg
MIM447	I	ctTTGGGGACGACTTAATGTTTTGTTGtttctagagggagataa
	II	aaCAACAAAACATTAAGTCGTCCCCAAagcttcggttcccctcg
MIM771	I	ctTGAGCCTCTGAAACGGTAGCCCTCtttctagagggagataa
	II	aaGAGGGCTACCGTTTCAGAGGCTCAagcttcggttcccctcg
MIM772	I	ctTTTTTCCTACATGACCGCCCATACtttctagagggagataa
	II	aaGTATGGGCGGTCATGTAGGAAAAAagcttcggttcccctcg
MIM773	I	ctTTTGCTTCCATAGACTTTTGTCTCtttctagagggagataa
	II	aaGAGACAAAAGTCTATGGAAGCAAAagcttcggttcccctcg
MIM774	I	ctTTGGTTACCCTAGTTATGGCCATCtttctagagggagataa
	II	aaGATGGCCATAACTAGGGTAACCAAagcttcggttcccctcg
MIM775	I	ctTTCGATGTCTTCGTGCAGTGCCAAtttctagagggagataa
	II	aaTTGGCACTGCACGAAGACATCGAAagcttcggttcccctcg
MIM776	I	ctTCTAAGTCTTTAGATATTGATGTTtttctagagggagataa
	II	aaAACATCAATATCTAAAGACTTAGAagcttcggttcccctcg
MIM777	I	ctTACGCATTGATAGATTTCGTTGCTtttctagagggagataa
	II	aaAGCAACGAAATCTATCAATGCGTAagcttcggttcccctcg
MIM778	I	ctTGGCTTGGTTATGAATGTACACCGtttctagagggagataa
	II	aaCGGTGTACATTCATAACCAAGCCAagcttcggttcccctcg

MIM779	I	ctTTCTGCTATGATGATGCTGCTCATtttctagagggagataa
	II	aaATGAGCAGCATCATCATAGCAGAAagcttcggttcccctcg
MIM780	I	ctTTTCTTCGTGTAGTATATCTGGCAtttctagagggagataa
	II	aaTGCCAGATATACTACACGAAGAAAagcttcggttcccctcg
MIM781	I	ctTTAGAGTTTTTAGATGGATACTTAtttctagagggagataa
	II	aaTAAGTATCCATCTAAAAACTCTAAagcttcggttcccctcg
MIM782	I	ctACAAACACCTATGAGGATGTTCTTtttctagagggagataa
	II	aaAAGAACATCCTCATAGGTGTTTGTagcttcggttcccctcg
MIM783	I	ctAAGCTTTGCTTAGAGTTCATGTTCtttctagagggagataa
	II	aaGAACATGAACTCTAAGCAAAGCTTagcttcggttcccctcg
MIM823	I	gaaccgaagctTGGGTGGTGAGGGTCATATAAGATtttctagagg
	II	cctctagaaaATCTTATATGACCCTCACCACCCAagcttcggttc
MIM824	I	gaaccgaagctTAGACCATTTAAAGTGAGAAGGGAtttctagagg
	II	cctctagaaaTCCCTTCTCACTTTAAATGGTCTAagcttcggttc
MIM828	I	gaaccgaagctTCTTGCTTAATTGATGAGTATTCCAtttctagagg
	II	cctctagaaaTGGAATACTCATCAATTAAGCAAGAagcttcggttc
MIM834	I	gaaccgaagctTGGTAGCAGTGGGAGCGGTGGTAAtttctagagg
	II	cctctagaaaTTACCACCGCTCCCACTGCTACCAagcttcggttc
MIM838	I	gaaccgaagctTTTTCTTCTATTGCTTCTTGCACAtttctagagg
	II	cctctagaaaTGTGCAAGAAGCAATAGAAGAAAAagcttcggttc
MIM841	I	gaaccgaagctTACGAGCCACAAGTTGAAACTGAAtttctagagg
	II	cctctagaaaTTCAGTTTCAACTTGTGGCTCGTAagcttcggttc
MIM847	I	gaaccgaagctTCACTCCTCTAAGTCTTCTTGATGtttctagagg
	II	cctctagaaaCATCAAGAAGACTTAGAGGAGTGAagcttcggttc
MIM858	I	gaaccgaagctTTCGTTGTCTAAAGTTCGACCTTgtttctagagg
	II	cctctagaaaCAAGGTCGAACTTTAGACAACGAAagcttcggttc
MIM859	I	gaaccgaagctTCGAACTCATAAGCTTTGGTGTCtttctagagg
	II	cctctagaaaGACACCAAAGCTTATGAGTTCGAagcttcggttc
MIMB	I	gaaccgaagctTTAGTAACAGATTCATCTTTGATTGGtttctagagg
	II	cctctagaaaCCAATCAAAGATGAATCTGTTACTAAagcttcggttc
MIMH	I	gaaccgaagctTCTCTCTGTTAAAGTGAAGTCAAatttctagagg
	II	cctctagaaaTTTGACTTCACTTTAACAGAGAGAagcttcggttc

Nucleotides complementary to the targeted miRNA are shown in upper case. *MIMB* and *MIMH* target the putative miRNAs candidates B and H, as described in [33].
