# Supplementary material for: A Collection of Target Mimics for Comprehensive Analysis of MicroRNA Function in Arabidopsis thaliana
Source: PLoS Genet. 2010 Jul 22;6(7):e1001031. doi: 10.1371/journal.pgen.1001031 (PMC2908682; doi:10.1371/journal.pgen.1001031)
Supplement: Table S2 — Oligonucleotide primers for qRT-PCR. (0.07 MB PDF) [file pgen.1001031.s005.pdf]

Supplementary Table 2. Oligonucleotide primers for qRT-PCR.

MiRNA	Target gene	Primer*	Sequence
**miR160**	*ARF10*	F	CGTTAAGCGGGTTAGTCCG
		R	CCTGAACCATAGCAGGATTC
**miR164**	*CUC1*	F	GAAGAGTTGTTGGGTCATGC
		R	CGAAATCAATCTGTCCCGATG
**miR165/6**	*PHB*	F	GTCTATGAGAATGGCCACATG
		R	GTAGGAGCATACATCTGCGTG
**miR169**	*HAP2C*	F	AACTCCGATAAACCGAGCC
		R	TCTTGGTCGTTCTTGTGATGTC
**miRs170/1**	*At3g60630*	F	GGATCTCAACACCTCCAGTG
		R	GAGACGGTGGTGATAATGATG
**miR172**	*AP2*	F	CGGGCAGCAGCAACATTGGTAGCGGA
		R	AGAGGAGGTTGGAAGCCATTTGTCTGC
**miR390/1**	*TAS3*	F	AAACTGCAGAGAAAAACGTCAACTTCTTTATTGAA
		R	AAAGAATCCTTCTTCGGCATTCCAGTTTC
**miR393**	*TIR1*	F	CTCTCTATCTGGCCTCTTGAC
		R	CAGGCATGTCAAATCGAGGAC
**miR394**	*At1g27340*	F	CTATCCACAGCACACTTTAC
		R	CACAGCATTGTAGGTTATCAG
**miR775**	*At1g53290*	F	GCGTTGTTAGTGGCGAAAAG
		R	CATCCTAGGTACGTCTGAG
**miR776**	*At5g62310*	F	CACTGGACTTCATGATTGAGC
		R	CCATTCAAGTACTCCATGACC

*F, forward; R, reverse.
